# Influenza Virus Samples, International Law, and Global Health Diplomacy

**DOI:** 10.3201/eid1401.070700

**Published:** 2008-01

**Authors:** David P. Fidler

**Affiliations:** * Indiana University School of Law, Bloomington, Indiana, USA

**Keywords:** Avian influenza, pandemic influenza, International Health Regulations, international law, World Health Organization, vaccines, surveillance, governance, biological resources, sovereignty, perspective

## Abstract

Indonesia’s decision to withhold samples of avian influenza virus A (H5N1) from the World Health Organization for much of 2007 caused a crisis in global health. The World Health Assembly produced a resolution to try to address the crisis at its May 2007 meeting. I examine how the parties to this controversy used international law in framing and negotiating the dispute. Specifically, I analyze Indonesia’s use of the international legal principle of sovereignty and its appeal to rules on the protection of biological and genetic resources found in the Convention on Biological Diversity. In addition, I consider how the International Health Regulations 2005 applied to the controversy. The incident involving Indonesia’s actions with virus samples illustrates both the importance and the limitations of international law in global health diplomacy.

## Abstract 2

An incident that involved withholding avian influenza virus samples illustrates the importance and limitations of international law in global health diplomacy.

On May 23, 2007, the World Health Assembly (WHA) adopted a resolution on sharing influenza viruses and promoting access to vaccines in connection with pandemic influenza preparedness (*1*). This resolution constituted the latest development in a controversy sparked by Indonesia’s decision to withhold influenza A (H5N1) samples from the World Health Organization (WHO) (*2*). The negotiations that produced WHA’s resolution involved complex international legal questions, which stimulated different answers from the parties involved. This article reviews this controversy and analyzes key international legal issues it generated.

## Indonesia’s Decision to Withhold Influenza A Virus (H5N1) Samples

This controversy began toward the end of 2006, when Indonesia decided not to share influenza A virus (H5N1) samples with WHO for risk assessment (e.g., surveillance) or risk management (e.g., vaccine development) purposes. Indonesia’s decision reportedly stemmed from its reaction to an Australian company’s development of an avian influenza vaccine derived from a virus strain that Indonesia provided to WHO (*3*). WHO’s acknowledgment that patents had been sought on modified versions of influenza (H5N1) samples shared through the Global Influenza Surveillance Network (GISN) without the consent of the countries that supplied the samples reinforced Indonesia’s discontent. Indonesia argued that this incident exposed inequities in the global influenza surveillance system. Developing countries provided information and virus samples to the WHO-operated system; pharmaceutical companies in industrialized countries then obtained free access to such samples, exploited them, and patented the resulting products, which the developing countries could not afford. Avian influenza’s spread and fears about pandemic influenza heightened this perceived inequity; experts argued that developing countries would have little access to vaccine for pandemic influenza without major changes in global vaccine production (*4**,**5*).

Indonesia’s action alarmed the global health community. Indonesia has been hit hard by avian influenza (*6*), so its cooperation in tracking the influenza virus (H5N1) was critical. Without access to Indonesia’s influenza strains, global surveillance was jeopardized, as was the refinement of diagnostic reagents and the development of intervention strategies, which depend on the information surveillance provides.

Regaining access to Indonesia’s samples motivated WHO to try to find a solution to the problem that Indonesia highlighted. In essence, Indonesia was making sample sharing for risk assessment dependent on action taken by WHO and industrialized countries to increase Indonesia’s access to influenza vaccines derived from samples it provided. Restarting sample sharing and improving vaccine access proved difficult and contentious. Before the WHA meeting in May 2007, negotiations between Indonesia and WHO did not produce agreement. For example, neither the Joint Statement issued by Indonesia and WHO in February 2007 (*7*) nor subsequent attempts to end the impasse succeeded (*8*). Independent efforts to increase vaccines access, such as the agreement of the United States and Japan in March 2007 to provide $18 million to 6 developing countries (Brazil, India, Indonesia, Mexico, Thailand, and Vietnam) to facilitate the building of vaccine-manufacturing capacity and of a vaccine stockpile (*9*), did not alter the stand-off.

## The World Health Assembly’s Resolution

Agreement at WHA was reached only through last-minute negotiations, which again illustrates the difficulties raised by Indonesia’s strategy to gain better access to influenza vaccines. The WHA resolution sets out a series of actions to achieve both “the timely sharing of viruses and specimens” in GISN and the promotion of “transparent, fair and equitable sharing of the benefits arising from the generation of information, diagnostics, medicines, vaccines and other technologies” (*1*). Most of the resolution consists of requests by WHO member states for the director-general to undertake activities designed to achieve fair and equitable sharing of benefits derived from influenza surveillance activities, especially access to vaccines (Table).

**Table:** Summary of actions that World Heath Organization member states requested of director-general

• To identify and propose, in consultation with member states, frameworks and mechanisms that aim to ensure fair and equitable sharing of benefits among all member states, taking strongly into consideration the specific needs of developing countries
• To establish, in consultation with member states, an international stockpile of vaccines for influenza (H5N1) or other influenza viruses of pandemic potential
• To formulate mechanisms and guidelines, in consultation with member states, aimed at ensuring fair and equitable distribution of pandemic influenza vaccines at affordable prices in the event of a pandemic to ensure timely availability of such vaccines to member states in need
• To mobilize financial, technical, and other appropriate support from member states, vaccine manufacturers, development banks, charitable organizations, private donors, and others to implement mechanisms and increase the equitable sharing of benefits as described in the resolution
• To convene an interdisciplinary working group to revise the terms of reference of WHO Collaborating Centers, H5 Reference Laboratories, and national influenza centers, devise oversight mechanisms, formulate draft standard terms and conditions for sharing viruses between originating countries and WHO Collaborating Centers, between the latter and third parties, and to review all relevant documents for sharing influenza viruses and sequencing data, based on mutual trust, transparency, and overriding principles
• To assure a member of the interdisciplinary working group consisting of 4 member states from each of the 6 WHO regions, taking into account balanced representation between industrialized and developing countries and including both experts and policymakers
• To convene an intergovernmental meeting to consider the reports by the director-general and by the interdisciplinary working group, which shall be open to all member states and regional economic organizations
• To commission an expert report on the patent issues related to influenza viruses and their genes, and report to the intergovernmental meeting
• To continue work with member states on the potential for conversion of existing biological facilities, such as those for the production of veterinary vaccines, so as to meet the standards for development and production of human vaccines, thereby increasing the availability of pandemic vaccines, and to enable them to receive vaccine seed strains
• To report on progress on implementation of the resolution to the World Health Assembly

Particularly important are the requests for the director-general to convene a) a working group to review, and propose reforms for, the sharing of influenza viruses and their use within and outside GISN; and b) an intergovernmental working group to consider progress being made toward the resolution’s goals, especially fair and equitable access to influenza vaccine for developing countries. These requests ensure that the linkage between virus sample sharing and equitable access to influenza vaccine remains prominent on the global health agenda for the foreseeable future.

The resolution reflects the current structure of global influenza governance (*10*). International sharing of influenza virus samples has occurred for decades within GISN (*11*). Although WHO and partners, such as the United Nations Children’s Fund (UNICEF) and GAVI Alliance, have increased developing-country access to childhood vaccines, mechanisms for increasing these countries’ access to influenza vaccines are weaker. Fears about avian influenza’s spread and the emergence of pandemic influenza highlighted the weakness of international efforts to increase vaccine availability in developing countries. The resolution attempts to build a multilateral process to address the lack of fair and equitable access for developing countries to pharmacologic benefits derived from the sharing of influenza virus samples. The resolution expresses a desire to craft a more equitable system of global influenza governance, the substantive elements of which remain to be negotiated.

## Political Dynamics of Influenza Virus Samples and Sovereignty over Biological Resources

The need to improve influenza vaccine access was recognized before this controversy (*4**,**5*), but Indonesia’s willingness to leverage control over virus samples to provoke more multilateral responses to the access problem changed the political dynamics of this issue. As typically happens when countries or international organizations challenge the status quo, the parties in this controversy framed their positions by using international law. This section analyzes how the stakeholders used international law to shape the debate. This incident illustrated the importance and limitations of international law in global health diplomacy.

By withholding samples, Indonesia asserted sovereignty over them because they originated within its territory. Despite controversies surrounding it, the principle of sovereignty remains a central tenet of international law (*12*). Traditionally, sovereignty holds that a state has authority and control over the people, resources, and activities within its territory (*12*). International law supplements sovereignty with the rule prohibiting states from intervening in each other’s domestic affairs (*12*). Limits on sovereignty arise when the state agrees to follow rules of international law found in treaties or customary international law.

In essence, Indonesia claimed that the samples are its sovereign property and do not constitute resources that other countries or the international community can access and use without Indonesia’s consent. This claim cut against the ethos and practice of sample sharing under which GISN had operated. This ethos and practice are based on accessing and analyzing influenza virus samples to produce accurate surveillance data, which inform development of interventions (e.g., vaccines).

Indonesia did not equate this ethos with an international legal obligation to engage in sharing that limited its sovereign rights over the samples. From a legal perspective, Indonesia’s arguments were plausible. GISN was not organized under treaty law, so no countries had treaty obligations to share samples. In addition, international law on infectious diseases applicable to Indonesia when this controversy began contained no obligations to share samples with WHO. The most relevant international legal rules, the International Health Regulations (IHRs) adopted by WHO in 1969 (IHR 1969), did not include influenza as a disease subject to the Regulations, nor did IHR 1969 require sharing of biological samples for the diseases covered (*13*).

Whether sharing obligations arose under customary international law when this controversy arose is also doubtful. To rise to the level of customary law, evidence must exist that states generally and consistently follow a practice out of a sense of legal obligation (*12*). GISN has, however, functioned without much, if any, reference to international law, making it difficult to establish that countries shared samples with WHO because they felt legally obligated to do so.

## Sovereignty Claims and the Application of Convention on Biological Diversity

In addition to exploiting basic principles of international law, Indonesia exploited precedents in other areas to bolster its sovereignty claims over the samples. Specifically, Indonesia borrowed from the international law developed to address biological diversity. The Convention on Biological Diversity (CBD) recognizes that countries have sovereign control of biological resources found within their territories (*14*). CBD defines biological resources to include “genetic resources, organisms or parts thereof, populations, or any other biotic component of ecosystems with actual or potential use or value for humanity” (article 2). Genetic resources are defined to mean “genetic material of actual or potential value”; genetic material means “any material of plant, animal, microbial or other origin containing functional units of heredity” (article 2). CBD further states that “the authority to determine access to genetic resources rests with the national governments and is subject to national legislation” (article 15.1). In addition, “access to genetic resources shall be subject to prior informed consent of the Contracting Party providing such resources” (article 15.5). Any access granted “shall be on mutually agreed terms” (article 15.4).

Indonesia’s claims that it controlled access to samples collected in its territory, that no use of such samples by other parties could occur without its prior informed consent, and that any use of such samples should produce benefits for Indonesia reflect the approach taken in CBD. Evidence that Indonesia framed the controversy by using these principles can be found in WHA’s 2007 resolution, which states that the Assembly “[r]ecogniz[es] the sovereign right of States over their biological resources” (preamble).

However, equating influenza virus samples with biological resources addressed by CBD raises questions that undermine Indonesia’s use of CBD. To begin, interpreting CBD to apply to pathogenic viruses may be contrary to CBD’s purpose. CBD was created, in part, to help developing countries rich in biological diversity control access to this diversity to conserve and manage it for sustainable development. Developing countries were concerned that corporate entities from industrialized countries were accessing their biological diversity and creating profitable products without the populations of these developing countries benefiting. Critics called this practice biopiracy (*15**,**16*).

Thus, the biological and genetic materials of primary CBD concern are indigenous resources in which governments, communities, and persons have invested time, effort, and resources to protect, cultivate, understand, and use. CBD provides that “States have sovereign rights over *their own biological resources*” (preamble [emphasis added]). In short, companies in the industrialized world were unjustly enriching themselves by profiting from previous efforts made in the developing country.

The avian influenza viruses affecting Indonesia are not the kind of biological and genetic resources that CBD sought to protect and regulate through the principles of sovereignty, prior informed consent, and mutual benefits from access and exploitation. These viruses invaded Indonesia; their presence and spread owes nothing to the investment, nurturing, and utilization of the Indonesian government or people. Rather than seeking to conserve this virus, the strategy is to contain and ultimately eradicate it. Applying CBD’s principles to influenza virus samples seems inappropriate given the difference between CBD’s object and purpose and the threat posed by influenza viruses.

State practice under CBD supports the conclusion that CBD does not apply to avian influenza virus. States parties to CBD have addressed avian influenza, not as a biological resource subject to CBD but as a threat to biological diversity. CBD discussions of avian influenza have considered its potential impact on wildlife, and the CBD process emphasized that surveillance is critical for combating avian influenza’s threat to biological diversity. Surveillance suffers without sharing information and samples of avian influenza viruses (*17*). Rather than protecting biological diversity, as mandated by CBD, Indonesia’s withholding virus samples from global surveillance efforts jeopardizes biological diversity in addition to population health.

Using CBD as a template in the context of influenza virus samples may be questionable on other grounds (*18*). The definitions of biological resources and genetic resources emphasize that the resources in question should be of actual or potential use or value for humanity. When these definitions are read in conjunction with CBD’s principles, this potential use or value for humanity is understood to derive from the protection, conservation, and sustainable use of the resources in question. CBD uses the principle of sovereignty as a regulatory instrument to achieve these goals. The use or value for humanity of influenza viruses comes from their widespread sharing for surveillance and vaccine development purposes because of the global threat such viruses pose. In this context, the principle of sovereignty central to the CBD approach is not a useful basis for facilitating timely and comprehensive sharing that global health governance requires.

## Virus Sharing and the Application of IHR 2005

One reason Indonesia stressed the CBD is that it provided a way to finesse the implications of the revised IHRs adopted by WHA in May 2005 (IHR 2005) (*19*), which provide that “[t]he provisions of the IHR shall not affect the rights and obligations of any State Party deriving from other international agreements” (article 57.1). Appeal to this rule begs the question raised by the first sentence of article 57.1, which states that “the IHR and other relevant international agreements should be interpreted so as to be compatible.” Thus, interpreting IHR 2005 became important in the controversy over influenza virus (H5N1) sharing. IHR 2005 is a treaty, “an international agreement concluded between States in written form and governed by international law” (*20*). This controversy represented an early test for how IHR 2005 would be interpreted and applied.

IHR 2005’s use proved complex for technical and substantive reasons. Technically, IHR 2005 had no binding force under international law until it officially entered into force on June 15, 2007. Thus, IHR 2005 created no international legal obligations for Indonesia with respect to the withholding of samples in the period before the Regulations entered into force. However, IHR 2005’s imminent entry into force made its substantive provisions relevant to the negotiations over Indonesia’s position on virus sharing.

Under international law, a state must refrain from acts that would defeat a treaty’s object and purpose when the state has expressed its consent to be bound by the treaty, pending the treaty’s entry into force (*20*). Indonesia had expressed its consent to be bound by IHR 2005 because it did not reject IHR 2005, or submit reservations to it, by the December 2006 deadline to do so. Thus, whether Indonesia’s decision to withhold samples constituted an act that would defeat the object and purpose of the IHR 2005 became a relevant question. Criticisms that Indonesia’s action fundamentally jeopardized global health security—the very object of IHR 2005 (*21*)—demonstrate that Indonesia could be considered in violation of its duty to not defeat the object and purpose of IHR 2005 before its entry into force.

This argument is supported by the claim that had IHR 2005 actually been in force, Indonesia would have violated its obligation to share samples. WHO Director-General Margaret Chan argued at the WHA meeting in May 2007 “that countries that did not share avian influenza virus would fail the IHR” (*22*). Addressing the credibility of these legal claims requires interpreting what IHR 2005 mandates States Parties to disclose and share with WHO. At least 2 differing interpretations exist. The first interpretation argues that IHR 2005 requires States Parties to share relevant biological samples as part of the duty to provide WHO with accurate and detailed public health information about all events that might constitute a public health emergency of international concern (PHEIC). Given that the spread of highly pathogenic influenza viruses is considered a PHEIC, the IHR 2005 mandates that States Parties provide WHO with samples for surveillance purposes without preconditions or expectations of benefits in return.

Supporting this interpretation is a WHA resolution adopted in May 2006, which called upon WHO member states “to comply immediately, on a voluntary basis, with provisions of the IHR 2005 considered relevant to the risk posed by avian influenza and pandemic influenza” (para. 1) (*23*). This resolution urged WHO member states “to disseminate to WHO collaborating centres information and relevant biological materials related to highly pathogenic avian influenza and other novel influenza strains in a timely and consistent manner” (para. 4[5]). The encouragement to share biological materials with WHO could be considered authoritative guidance from WHO’s highest policymaking body about the scope of the obligation to share public health information with WHO with respect to all events that might constitute a PHEIC.

This interpretation was succinctly stated by the US delegation to WHA: “All nations have a responsibility under the revised IHRs to share data and virus samples on a timely basis and without preconditions. The United States wishes to be clear that our view is that withholding influenza viruses from GISN greatly threatens global public health and will violate the legal obligations we have all agreed to undertake through our adherence to IHRs” (*24*).

Even though IHR 2005 never expressly requires the sharing of biological samples, a good faith interpretation of IHR 2005 in light of its object and purpose acknowledges a duty to share such samples for surveillance purposes. An opposite interpretation could lead to a manifestly absurd or unreasonable result, which treaty interpretation principles do not support. This interpretation of IHR 2005 also is compatible with CBD because IHR 2005 requires sample sharing for risk assessment purposes, not risk management activities. Thus, the sharing mandate in IHR 2005 does not preclude WHO and its member states from crafting arrangements to improve access to benefits, such as vaccines, derived from samples shared for surveillance purposes.

The second interpretation comes to the opposite conclusion. This position asserts that, under principles of treaty interpretation, IHR 2005 does not require States Parties to share biological samples with WHO. The first principle of treaty interpretation is that a treaty shall be interpreted in good faith in accordance with the ordinary meaning to be given to the terms of the treaty in their context and in light of its object and purpose (*20*). IHR 2005 requires States Parties to provide WHO with “public health information” about events that may constitute a PHEIC (article 6). IHR 2005 does not define what “public health information” means, so its meaning has to be discerned through treaty interpretation principles. The second interpretation holds that the ordinary meaning of “information” encompasses knowledge and facts (*25*) but does not include biological samples.

The second interpretation maintains that IHR 2005, its negotiations, and the WHA resolutions of 2006 and 2007 support it. Nowhere does IHR 2005 contain any express requirement to share samples of biological materials. The only provision that refers to biological substances provides that: “States Parties shall, subject to national law and taking into account relevant international guidelines, facilitate the transport, entry, exit, processing and disposal of biological substances and diagnostic specimens, reagents and other diagnostic materials for verification and public health response purposes under these Regulations” (article 46). The use of “biological substances” here suggests that the negotiators considered this concept separate from “public health information.”

The provision that contains the duty to communicate public health information to WHO about a reported event also contains a list of things that fall within this obligation: case definitions, laboratory results, source and type of risk, number of cases and deaths, conditions affecting the spread of disease, and the health measures used (article 6.2). This list refers to things that would fall within the ordinary meaning of “information” and contains nothing that could be considered biological samples, substances, or specimens. The absence of express reference to biological samples is particularly telling in light of the fact that WHO and its member states were, at the time IHR 2005 was being negotiated, aware of concerns about the failure of countries to share samples of pathogens of global concern (e.g., the severe acute respiratory syndrome virus, the influenza [H5N1] virus) for surveillance and other purposes.

Similarly, an earlier negotiating text included the following provision: “In the context of a suspected intentional release of a biological, chemical or radionuclear agent, States shall immediately provide to WHO all relevant public health information, materials and samples, for verification and response purposes” (*26*). Here again, the negotiators used “public health information” and “samples” as distinct terms. Further, this provision does not appear in IHR 2005. Even if it had so appeared, it would have underscored that sharing samples was only required in connection with suspected intentional use of a biological, chemical, or radionuclear agent, which does not include the natural emergence of avian or pandemic influenza.

WHA resolutions of 2006 and 2007 also support this interpretation. The 2006 resolution on early compliance with IHR 2005 with respect to influenza threats urges WHO member states to disseminate to WHO “information *and* relevant biological materials” (*23*) (emphasis added), which further demonstrates that WHO member states consider public health information and biological materials different, not equivalent, terms. WHA’s 2007 resolution uses the same language in recalling the 2006 resolution’s urging of WHO member states to disseminate information *and* relevant biological materials (*1*). This interpretation is also compatible with CBD because it leaves the decision whether to share biological samples in the hands of the state party in which the samples originate.

## Beyond Differing Treaty Interpretations and the WHA Resolution

Stepping back from the differing treaty interpretations, Indonesia’s actions exposed ambiguity in a critical aspect of IHR 2005 on the eve of its entry into force. The WHA’s 2007 resolution did not resolve this controversy because, on this question, its provisions provide no clear answer. The resolution reaffirms the obligations of States Parties under IHR 2005 and the sovereign right of states over their biological resources, a key principle in CBD. The bargain that underpins the resolution has, however, established the utility of countries’ withholding samples to force WHO and industrialized countries to address neglected aspects of global influenza governance. Dueling treaty interpretations may matter less than the old legal adage that possession of property in dispute is nine-tenths of the law. When possession is cloaked in the principle of sovereignty, those who require access to the property have to come to terms with the need to bargain for it.

Conceptually, the WHA’s 2007 resolution seeks to achieve equitable use of influenza virus samples. Such equitable use encompasses timely sharing of samples for global surveillance and more effort to ensure that developing countries share in the benefits of knowledge and technologies derived from the samples, especially influenza vaccines. Equitable use has not occurred because sharing influenza virus samples proves easier than producing equitable access to technologies derived from the knowledge produced by surveillance. The resolution itself obviously does not produce equitable use, but it establishes a WHO-based process for moving global health diplomacy in this direction. The resolution is a general blueprint for building new global governance mechanisms on equitable use of influenza samples. This blueprint is, however, technically limited to influenza virus sharing and vaccine development, and its creation raises questions about governance of the sharing of samples of other pathogens of global concern and of benefits derived from such samples.

WHO and its member states had started the process described in the resolution by, among other things, meeting in Singapore in July 2007 and scheduling another intergovernmental session in November 2007. The meeting in Singapore did not produce consensus, and Indonesia continued to withhold the samples (*27*). In reporting on the Singapore meeting, Branswell observed that many feared the talks would follow Indonesia’s lead and produce “a system where countries would exercise sovereign rights over viruses or bacteria found within their borders, seeking quid pro quos from vaccine makers or assessing the potential for gain before co-operating with global health authorities to squelch new disease threats like SARS.” (*28*) Media reported in September 2007 that Indonesia had shared some virus samples with WHO related to 2 fatal influenza (H5N1) cases in Bali (*29*), but this action did not mean that Indonesia had abandoned or repudiated the position it had staked out on virus sharing and access to vaccine. Thus, as of this writing, the fundamental issues at the heart of this controversy, including the international legal questions analyzed in this article, had not been resolved.

Whether the process sketched in WHA’s resolution produces an effective multilateral regime for equitable use remains to be seen. The process itself is not legally binding because WHA resolutions do not have the force of international law (*30*). The agreement to create this process will perpetuate legal disagreements about sovereignty, CBD, IHR 2005, and other legal issues (e.g., intellectual property rights) because neither side currently has an interest in having the legal questions definitively answered. Instead, constructive legal ambiguity informs the political willingness of countries to shoulder the equitable use responsibilities the WHA resolution envisions.
